# Predictive Role of Admission Venous Lactate Level in Patients with Upper Gastrointestinal Bleeding: A Prospective Observational Study

**DOI:** 10.3390/jcm11020335

**Published:** 2022-01-11

**Authors:** Marcin Strzałka, Marek Winiarski, Marcin Dembiński, Michał Pędziwiatr, Andrzej Matyja, Michał Kukla

**Affiliations:** 1 2nd Department of General Surgery, Faculty of Medicine, Jagiellonian University Medical College, 31-008 Kraków, Poland; marek.winiarski@uj.edu.pl (M.W.); m.dembinski@uj.edu.pl (M.D.); michal.pedziwiatr@uj.edu.pl (M.P.); andrzej.matyja@uj.edu.pl (A.M.); 2 Department of Endoscopy, University Hospital, 30-688 Kraków, Poland; michal.kukla@uj.edu.pl; 3 Department of Internal Medicine and Geriatrics, Faculty of Medicine, Jagiellonian University Medical College, 31-008 Kraków, Poland

**Keywords:** upper gastrointestinal bleeding, venous lactate, prediction of clinical outcomes, peptic ulcer, esophageal varices

## Abstract

Upper gastrointestinal bleeding (UGIB) is one of the most common emergencies. Risk stratification is essential in patients with this potentially life-threatening condition. The aim of this prospective study was to evaluate the usefulness of the admission venous lactate level in predicting clinical outcomes in patients with UGIB. All consecutive adult patients hospitalized due to UGIB were included in the study. The clinical data included the demographic characteristics of the observed population, etiology of UGIB, need for surgical intervention and intensive care, bleeding recurrence, and mortality rates. Venous lactate was measured in all patients on admission. Logistic regression analyses were used to calculate the odds ratios (OR) of lactate levels for all outcomes. The receiver operating characteristic (ROC) curve was used to determine the accuracy of lactate levels in measuring clinical outcomes, while Youden index was used to calculate the best cut-off points. A total of 221 patients were included in the study (151M; 70F). There were 24 cases of UGIB recurrence (10.8%), 19 patients (8.6%) required surgery, and 37 individuals (16.7%) required intensive care. Mortality rate was 11.3% (25 cases). The logistic regression analysis showed statistically significant association between admission venous lactate and all clinical outcomes: mortality (OR = 1.39, 95%CI: 1.22–1.58, *p* < 0.001), recurrence of bleeding (OR = 1.16, 95%CI: 1.06; 1.28, *p* = 0.002), surgical intervention (OR = 1.17, 95%CI: 1.06–1.3, *p* = 0.002) and intensive care (OR = 1.33, 95%CI: 1.19–1.5, *p* < 0.001). The ROC curve analysis showed a high predictive value of lactate levels for all outcomes, especially mortality: cut-off point 4.3 (AUC = 0.82, 95%CI: 0.72–0.92, *p* < 0.001) and intensive care: cut-off point 4.2 (AUC = 0.76, 95%CI: 0.66–0.85, *p* < 0.001). Admission venous lactate level may be a useful predictive factor of clinical outcomes in patients with UGIB.

## 1. Introduction

Acute upper gastrointestinal bleeding (UGIB) is considered as one of the most common emergencies and reasons of urgent hospitalization. The incidence of acute upper gastrointestinal hemorrhage ranges from 40 up to 150 cases a year in 100,000 population. Despite the continuous advances in medical knowledge and new methods of treatment, it is still considered as a potentially life-threatening condition with mortality rates from 8% up to 15% [1].

Proper risk evaluation and prediction of therapy results are essential in patients with UGIB. Several scoring systems have been created to predict clinical outcomes associated with UGIB, but none of these scoring systems used admission venous lactate level as a predictive factor of treatment results [1,2]. Existing scores with proven predictive value in patients with UGIB such as Rockall, Glasgow-Blatchford, or AIMS65 are often difficult to calculate at the patient’s bed, and thus not practical [1,2].

Lactate level has been proposed to use in the prediction of disease severity and risk estimation in many different illnesses [3]. Lactate measurements were considered as an important predictor especially in case of mortality. Some recent studies have suggested that lactate level is associated with prognosis also in patients with upper gastrointestinal bleeding [4,5,6].

However, our knowledge concerning the role of lactate level on admission in predicting clinical outcomes in patients with UGIB is still changing and incomplete [2,5,7], partially because of the retrospective character of the previous studies. It also seems important to check the universality of the predictive value of lactate level measurement in UGIB of various etiologies.

The aim of this prospective observational study was to evaluate the usefulness of the admission venous lactate level in predicting clinical outcomes in hospitalized patients with symptoms of acute UGIB. The end points determining clinical results in this group of patients were: in-hospital mortality, recurrence of bleeding, need for surgical treatment, and intensive therapy. This study aimed also to check the usefulness and universality of the predictive value of admission venous lactate measurements in UGIB of various etiologies.

## 2. Materials and Methods

All consecutive adult patients with symptoms of acute upper gastrointestinal bleeding admitted to the hospital from 1st January 2018 to 31st December 2019 were included in the study.

The clinical data was collected prospectively during the study period. It included the demographic characteristics of the observed population (number of patients, their age, and sex), etiology of UGIB, endoscopic treatment, need for surgical intervention, and intensive care unit (ICU) therapy, bleeding recurrence rates and in-hospital mortality rates. Patients under 18 years of age with symptoms of acute UGIB were excluded from the study.

All patients in the analyzed group were diagnosed and treated according to the present guidelines [1,8,9]. On admission, clinical examination with measurement of arterial blood pressure, pulse rate, and oxygen saturation was performed. In all cases, intravenous catheterization was done and blood samples for laboratory tests including lactate level were taken. Adequate intravenous fluid resuscitation was performed. All patients with symptoms of acute upper gastrointestinal bleeding received the initial dose of pantoprazole (80 mg i.v.) before the endoscopic procedure. Urgent gastroscopy with the application of endoscopic therapy was performed in all cases of UGIB during the first six hours after admission to hospital. Continuous intravenous infusion of pantoprazole (8 mg/h) was used in cases of non-variceal UGIB during 48–72 h after endoscopic treatment. Continuous intravenous infusion of somatostatin was administered in patients with hemorrhage caused by esophageal varices for 48–72 h after gastroscopy. Medication changes for oral pantoprazole (40 mg twice a day) or oral propranolol (10 mg three times a day) usually followed the return to normal diet. Surgical treatment of UGIB was dedicated to patients with unsuccessful endoscopic therapy, either primary or secondary (in case of recurrence of hemorrhage).

Criteria for patient admission to the intensive care unit were strictly defined and in line with current guidelines [10]. Patients with UGIB were qualified for the intensive care unit treatment when they had clinical instability, were at high risk for imminent decline or required care involving specialized competency of intensive care unit staff.

Venous lactate level was measured in all patients on admission as a part of the point of care testing (POCT). All measurements were performed with the application of the Radiometer ABL 800 FLEX analyzer (Radiometer Medical ApS, Denmark).

Continuous variables in the analyzed materials were expressed as the mean and standard deviation (SD). Categorical variables were summarized in terms of numbers and percentages. The Kolmogorov–Smirnov test was used to compare the mean values of the parameters. Student’s *t*-test was used for calculations in case of normal distribution of variables. The Mann–Whitney U test was used when they were not normally distributed. Logistic regression analyses were used to calculate the odds ratio (OR) of lactate admission level for all outcome parameters, with 95% confidence intervals (CIs). By running logistic regression models, it was possible to evaluate if lactate levels were significantly associated with recurrence of bleeding, surgical intervention, intensive care unit treatment, and in-hospital mortality. The receiver operating characteristic (ROC) curve was used to determine the accuracy of admission venous lactate levels in measuring clinical outcomes. The Youden index was calculated to determine the proposed cut-off point in each case, where the highest sensitivity and specificity of the ROC curve were taken. For all analyses, a *p* value < 0.05 was considered statistically significant. STATISTICA 13 PL software (StatSoft Polska Sp. z o.o., Kraków, Poland) was used to perform all statistical analyses.

## 3. Results

There were 221 patients including 151 males (68%) and 70 females (32%) in the analyzed group. The mean patient age was 63.5 ± 17.8 years (range: 19–86). Mean age of men was 61.6 ± 17.8 years (range: 22–84) and women 67.5 ± 17.3 years (range: 19–86).

There were 183 cases (83%) of non-variceal UGIB (mean age 65.3 ± 17.6 years) including 121 males (66%) and 62 females (34%). The subgroup of patients with variceal bleeding consisted of 38 patients (mean age 54.6 ± 16.5 years) including 30 males (79%) and 8 females (21%). Both subgroups of patients with UGIB did not differ in terms of gender distribution. Demographics of UGIB patients are summarized in Table 1.

The patients with non-variceal bleeding were older than the patients with variceal hemorrhage (*p* < 0.001). Both compared patient subgroups did not differ significantly in the frequency of tarry stools and syncope. In contrast, patients with variceal bleeding were more likely to have hematemesis, with lower mean systolic blood pressure, and higher mean heart rate. All these differences were statistically significant. Compared subgroups of patients did not differ significantly in the incidence of kidney disease, diabetes, and malignancies. However, a statistically significant difference occurred in the incidence of heart and liver diseases (Table 1).

The average venous lactate level measured on admission to hospital in all patients with UGIB was 3.54 ± 3.46 mmol/L. It was higher in the subgroup of patients with variceal (5.22 ± 5.60 mmol/L) compared to non-variceal bleeding (3.19 ± 2.71 mmol/L), but the difference was on the threshold of statistical significance (*p* = 0.06). The results of selected laboratory tests in all patients with UGIB and in the compared subgroups are presented in Table 1.

The most common etiology of UGIB was duodenal ulcer (36.2%), followed by gastric ulcer (24.9%), esophageal or stomach varices (17.2%), Mallory-Weiss syndrome (8.1%), erosive gastritis (5.4%), Dieulafoy lesion (2.3%), esophagitis (1.8%), gastric tumors (1.8%), angiodysplasia (1.4%) and erosive duodenitis (0.9%). The details associated with UGIB etiology are presented in Table 2.

There were 24 cases of UGIB recurrence (10.8%), 19 patients (8.6%) required surgical therapy. A total of 37 individuals (16.7%) were treated in the intensive care unit. In-hospital mortality rate in the analyzed group was 11.3% (25 cases).

Subgroups of patients with variceal and non-variceal bleeding did not differ statistically in terms of in-hospital mortality, bleeding recurrence rate, need for surgical treatment, and intensive care (Table 1).

### 3.1. Mortality

The logistic regression analysis showed that there was a statistically significant association between admission venous lactate level and in-hospital mortality (OR = 1.39, 95%CI: 1.22–1.58, *p* < 0.001). That means that a 1 mmol/L increase of admission lactate level was associated with 39% increased odds of in-hospital mortality in patients with UGIB (Table 3).

**Figure 1:**
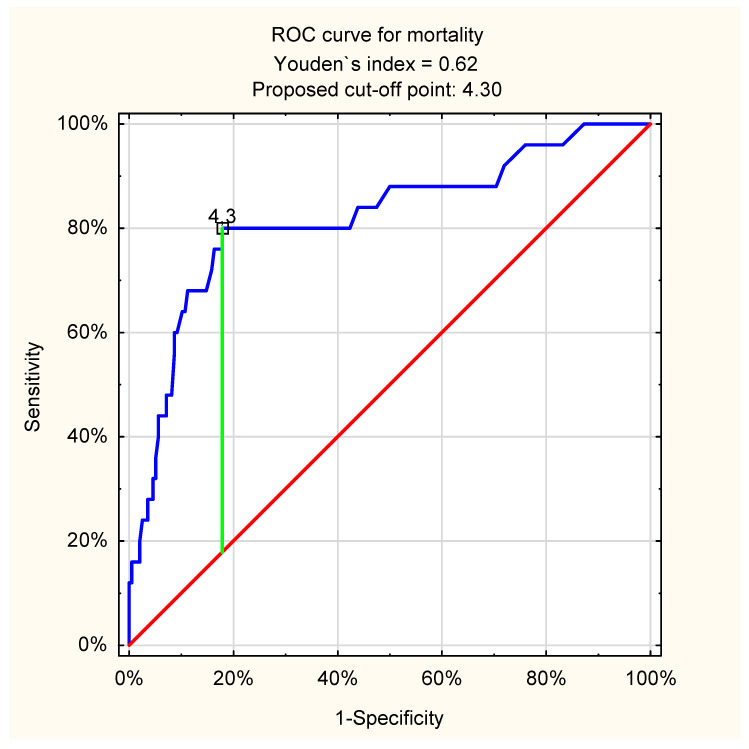
ROC curve for admission venous lactate level and in-hospital mortality in all patients with UGIB (proposed cut-off point = 4.3 mmol/L; sensitivity = 0.80; specificity = 0.82; accuracy = 0.82; AUC = 0.82 (95%CI: 0.72–0.92); *p* < 0.001).

The ROC curve analysis showed a high predictive value of lactate levels for mortality in all patients with UGIB, regardless of its etiology: cut-off point 4.3 mmol/L; AUC = 0.82 (95%CI: 0.72–0.92, *p* < 0.001). The details associated with ROC curve analysis in all patients with UGIB are presented in Figure 1.

**Figure 2:**
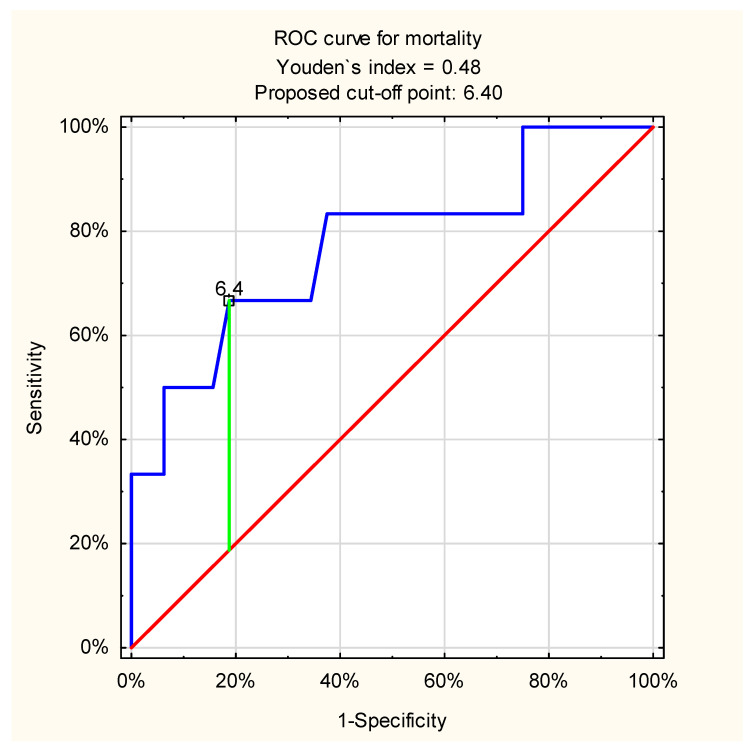
ROC curve for admission venous lactate level and in-hospital mortality in patients with variceal UGIB (proposed cut-off point = 6.4 mmol/L; sensitivity = 0.67; specificity = 0.81; accuracy = 0.79; AUC = 0.78 (95%CI: 0.55–1.0); *p* = 0.01).

The ROC curve analysis for admission venous lactate level and in-hospital mortality in patients with variceal UGIB showed a slightly weaker but still acceptable discriminatory ability of the test: cut-off point 6.4 mmol/L; AUC = 0.78 (95%CI: 0.55–1.0, *p* = 0.01) (Figure 2).

**Figure 3:**
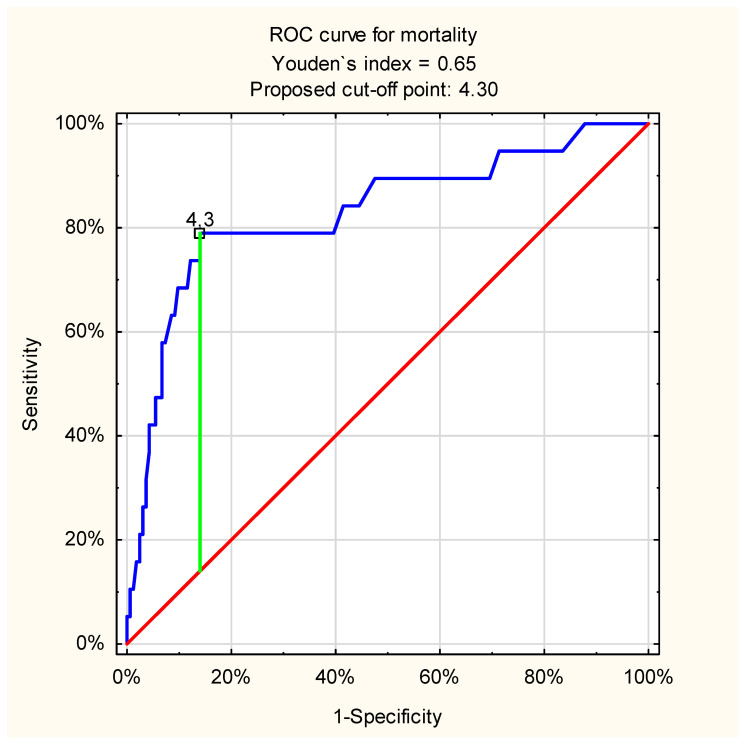
ROC curve for admission venous lactate level and in-hospital mortality in patients with non-variceal UGIB (proposed cut-off point = 4.3 mmol/L; sensitivity = 0.79; specificity = 0.86; accuracy = 0.85; AUC = 0.83 (95%CI: 0.72–0.94); *p* < 0.001).

In turn, ROC curve analysis indicated an excellent value of this parameter in predicting mortality in patients with non-variceal UGIB: cut-off point = 4.3 mmol/L, AUC = 0.83 (95%CI: 0.72–0.94, *p* < 0.001) (Figure 3).

**Figure 4:**
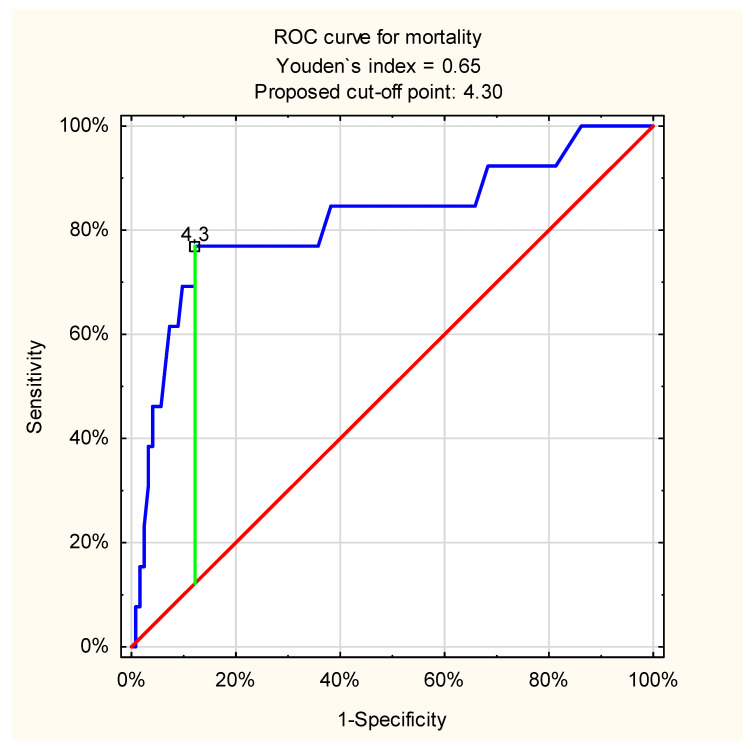
ROC curve for admission venous lactate level and in-hospital mortality in patients with bleeding peptic ulcers (proposed cut-off point = 4.3 mmol/L; sensitivity = 0.77; specificity = 0.88; accuracy = 0.87; AUC = 0.82 (95%CI: 0.67–0.96), *p* < 0.001).

The most frequent cause of the UGIB was bleeding peptic ulcer. ROC curve analysis also showed excellent discriminatory ability of admission venous lactate level and in-hospital mortality in this etiologically homogeneous and important patient subgroup: proposed cut-off point = 4.3 mmol/L, AUC = 0.82 (95%CI: 0.67–0.96, *p* < 0.001) (Figure 4).

### 3.2. Recurrence of Bleeding

The logistic regression models showed that there was a statistically significant association between admission venous lactate level and recurrence of bleeding (OR = 1.16, 95%CI: 1.06–1.28, *p* = 0.002). That means that a 1 mmol/L increase of admission lactate level was associated with 16% increased odds of hemorrhage recurrence in patients with UGIB.

ROC curve analysis showed only poor accuracy of this test in predicting recurrence in all patients with UGIB: cut-off point 2.2 mmol/L; AUC = 0.64 (95%CI: 0.53–0.76, *p* = 0.01).

### 3.3. Surgical Treatment

The logistic regression analysis showed that there was a statistically significant association between admission venous lactate level and risk of surgical treatment (OR = 1.17, 95%CI: 1.06–1.3, *p* = 0.002). That means that a 1 mmol/L increase of admission lactate level was associated with 17% increased odds of surgical intervention in patients with UGIB.

ROC curve analysis for admission venous lactate level and need of operation in all patients with UGIB showed acceptable discriminatory ability of the test: cut-off point = 3.1 mmol/L; AUC = 0.71 (95%CI: 0.58–0.83, *p* = 0.001).

### 3.4. Intensive Care

**Figure 5:**
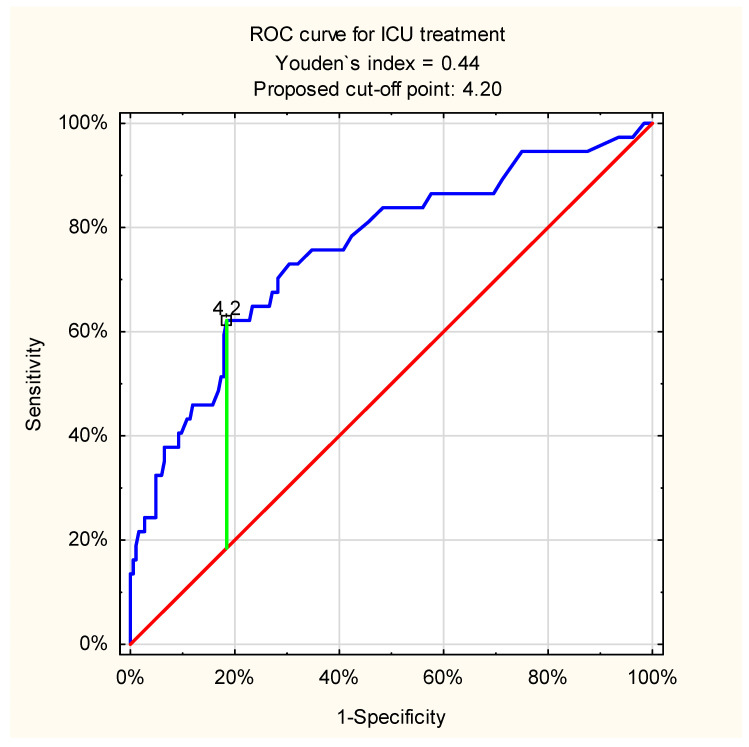
ROC curve for admission venous lactate level and need for ICU treatment in all patients with UGIB (proposed cut-off point = 4.2 mmol/L; sensitivity = 0.62; specificity = 0.82; accuracy = 0.78; AUC = 0.76 (95%CI: 0.66–0.85); *p* < 0.001).

The logistic regression models showed that there was a statistically significant association between admission venous lactate level and need for intensive care unit treatment, OR = 1.33 (95%CI: 1.19–1.50, *p* < 0.001). That means that a 1 mmol/L increase of admission lactate level was associated with 33% increased odds of intensive care unit therapy in patients with UGIB. ROC curve analysis in case of intensive care need in all patients with UGIB showed acceptable discriminatory ability of the test: cut-off point = 4.2 mmol/L; AUC= 0.76 (95%CI: 0.66–0.85, *p* < 0.001) (Figure 5).

Detailed results of logistic regression analysis in all patients and in the subgroups are presented in Table 3.

## 4. Discussion

Results of our study indicate that admission venous lactate measurement allows for the prediction of in-hospital mortality, recurrence of bleeding, and need for surgical treatment, or intensive care in patients with UGIB.

Lactic acid is an organic compound which in the form of its conjugate base called lactate plays an important role in some biochemical processes [3]. Lactate concentration increases in the body cells in case of anaerobic energy production. The transformation of glucose to lactate, called anaerobic glycolysis, occurs in case of limited amounts of oxygen, in other words, when aerobic energy production is insufficient or unavailable. This can be observed in a lot of conditions associated with severe illness. One of the examples of increased lactate production is hypovolemia. It leads to peripheral vasoconstriction and centralization of circulation to keep perfusion of vitally important organs, such as heart, brain, and lungs. However, body tissues with insufficient amounts of oxygen still require adenosine triphosphate (ATP) to keep proper function. They try to maintain homeostasis by changing their metabolic pathways from aerobic to anaerobic ones. Because of this, increased lactate concentration can be considered as a by-product of intracellular anaerobic energy production processes.

Lactate level measurement has been proposed to use in the prediction of illness severity and risk of mortality in many pathological processes. There were among them such clinically important conditions as: sepsis, trauma, cancer, poisoning, and pediatric cardiac disease [11].

Hyperlactatemia may be also caused by the decrease in circulating blood volume and subsequent tissue hypoxia in patients with acute upper gastrointestinal bleeding [4,5,6].

It is important that increased lactate production can develop even before the onset of other typical clinical signs of decreased tissue perfusion, such as hypotension [11]. Therefore, lactate measurements seem to be helpful also in patients with acute UGIB. Early identification of high-risk patients may help to indicate those requiring more rapid interventions, for example, urgent endoscopic treatment. Precise and fast recognition of severely ill patients with symptoms and signs of acute UGIB may allow to prevent poor clinical outcomes.

At least three lactate parameters can be measured and compared in patients with acute UGIB including: initial, maximal, and average lactate levels.

Recently, Korean authors reported that there are no significant differences of predicting power between the above three lactate parameters in patients with non-variceal UGIB, and the single admission lactate level test is supposed to be the most practical and useful [7].

It was also demonstrated that elevated initial serum lactate level was associated with mortality in patients with non-variceal UGIB similar to severe sepsis [11,12].

Recently, Stokbro et al. in their retrospective cohort study suggested that arterial lactate does not predict outcome better than existing risk scores in patients admitted to hospital with upper gastrointestinal bleeding [2]. However, on the other hand, it is much easier for the physician in everyday practice to use one simple measurement result than to calculate sometimes complicated scores.

Results of the current study showed that there is a statistically significant association between admission venous lactate level and all important clinical outcomes including: in-hospital mortality, recurrence of bleeding, need of surgical intervention, and intensive care unit treatment. Every 1-point increase in lactate level conferred a 1.39-fold increase in the odds of in-hospital mortality, a 1.33-fold increase in the odds of ICU treatment, and a 1.17-fold increase in the odds of surgical intervention. The correlation between admission venous lactate and UGIB recurrence was weaker, but still statistically significant.

It seems that the measurement of venous lactate level on admission to hospital may be a proper and universal test allowing for easy triage of patients with UGIB in emergency departments. The high predictive power of this test deserves special emphasis in all patients with UGIB, as well as in subgroups of non-variceal and ulcer bleeding. In the latter two clinically relevant subgroups, the correlation between high lactate concentration on admission and in-hospital mortality is particularly apparent.

Lactate levels are elevated in cirrhotic patients and they seem to increase with the severity of liver cirrhosis [13]. In our study, the mean lactate level on admission to hospital was higher in patients with variceal bleeding compared to the non-variceal bleeding group. It seems that in the case of variceal bleeding, it may be associated with chronic liver damage and insufficiency as well as with more rapidly developing and more severe hypovolemia resulting from usually massive bleeding.

Higher levels of lactate in patients with UGIB upon admission to hospital may indicate more severe metabolic disorders and more severe hypovolemia which increases mortality in this group. On the other hand, the ability to easily identify the most at-risk UGIB patients is of great value for the emergency department physician. It seems that the measuring of lactate levels on admission to the hospital may facilitate triage and indicate the need to adequately protect patients with UGIB symptoms regardless of proper bleeding control.

According to the authors’ best knowledge, this paper is the first prospective observational study evaluating the role of the admission venous lactate level in predicting clinical outcomes in hospitalized patients with symptoms of UGIB. The results of this study indicate the important predictive role of initial venous lactate testing in cases of this common emergency. In addition, nowadays lactate measurements are inexpensive and can be performed very quickly as a part of point of care testing.

The limitations of this study are associated with a relatively small number of cases in the subgroup of variceal bleeding, which can affect the results of all patients with acute UGIB. In addition, the severity of liver disease has not been carefully evaluated in patients with variceal bleeding. It should also be noted that advanced liver failure may be an independent cause of elevated blood lactate levels.

This study encourages further investigation of the predictive role of admission lactate level in cases of acute UGIB. Developing scoring systems for acute UGIB incorporating serum lactate and other biomarkers can be also beneficial for predicting outcomes. The value of serial measurements of serum lactate levels for predicting outcomes in acute UGIB and the role of using admission venous lactate to help in the triage of patients with acute UGIB should be investigated in future studies.

## 5. Conclusions

Predictive role of admission venous lactate level in patients with acute UGIB seems to be important. Results of our study indicate that this cheap, available, and useful single parameter allows for the prediction of in-hospital mortality, recurrence of bleeding, and need for surgical treatment, or intensive care in patients with UGIB.

Other prospective studies including large samples are necessary to assess precisely the impact of admission venous lactate level on clinical outcomes in acute UGIB before it is recommended for routine use.

## Figures and Tables

**Table 1:** Characteristics of patients hospitalized for upper gastrointestinal bleeding (data presented as numbers, percentages or means ± SD).

Parameters	All Patients with UGIB	Non-Variceal UGIB	Variceal UGIB	*p*-Value
*n* (%)	221 (100)	183 (83)	38 (17)	
males, *n* (%)	151 (68)	121 (66)	30 (79)	0.122
females, *n* (%)	70 (32)	62 (34)	8 (21)	
age (years) ± SD	63.5 ± 17.8	65.3 ± 17.6	54.6 ± 16.5	<0.001
symptoms				
syncope, *n* (%)	199 (90)	164 (90)	35 (92)	0.641
melena, *n* (%)	210 (95)	174 (95)	36 (95)	0.929
hematemesis, *n* (%)	174 (78)	136 (74)	38 (100)	<0.001
systolic blood pressure (mmHg) ± SD	104 ± 20	106 ± 20	98 ± 17	0.031
heart rate (n/min) ± SD	101 ± 22	100 ± 23	109 ± 16	0.019
comorbidities				
heart disease, *n* (%)	77 (34.8)	72 (39.3)	5 (13.2)	0.002
liver disease, *n* (%)	80 (36.2)	43 (23.5)	37 (97.4)	<0.001
kidney disease, *n* (%)	49 (22.2)	44 (24.0)	5 (13.2)	0.142
diabetes mellitus, *n* (%)	40 (18.1)	35 (19.1)	5 (13.2)	0.385
cancer, *n* (%)	24 (10.9)	23 (12.6)	1 (2.6)	0.073
blood test results				
hemoglobin (g/dL) ± SD	8.3 ± 2.7	8.3 ± 2.7	8.2 ± 2.7	0.968
lactate (mmol/L) ± SD	3.54 ± 3.46	3.19 ± 2.71	5.22 ± 5.60	0.063
albumin (g/dL) ± SD	30.2 ± 6.1	30.6 ± 6.0	28.3 ± 6.3	0.012
INR ± SD	1.60 ± 1.45	1.60 ± 1.57	1.59 ± 0.58	0.980
APTT (s) ± SD	37.6 ± 24.2	36.8 ± 24.4	41.4 ± 23.4	0.001
creatinine (µmol/L) ±SD	121.2 ± 102.4	125.5 ± 103.3	100.5 ± 96.3	0.047
glucose (mmol/L) ± SD	8.3 ± 3.8	8.2 ± 3.4	8.9 ± 5.5	0.617
treatment results				
rebleeding, *n* (%)	24 (10.8)	16 (8.7)	6 (15.8)	0.187
ICU treatment, *n* (%)	37 (16.7)	31 (16.9)	6 (15.8)	0.863
operation, *n* (%)	19 (8.6)	17 (9.3)	2 (5.3)	0.420
mortality, *n* (%)	25 (11.3)	19 (10.4)	6 (15.8)	0.338

**Table 2:** Etiology of UGIB in the analyzed patients.

Etiology of UGIB	Number of Patients	Percentage
duodenal ulcer	80	36.2%
gastric ulcer	55	24.9%
esophageal varices	38	17.2%
Mallory-Weiss syndrome	18	8.1%
erosive gastritis	12	5.4%
Dieulafoy lesion	5	2.3%
esophagitis	4	1.8%
gastric tumors	4	1.8%
angiodysplasia	3	1.4%
erosive duodenitis	2	0.9%
overall	221	100%

**Table 3:** Logistic regression analysis results of admission venous lactate levels for different clinical outcomes in all patients with UGIB and in the subgroups.

Patient Groups	Outcomes	*p* Value	OR	95%CI
all patients with UGIB	mortality	<0.001	1.39	(1.22–1.58)
	rebleeding	0.002	1.16	(1.06–1.28)
	operation	0.002	1.17	(1.06–1.30)
	ICU treatment	<0.001	1.33	(1.19–1.50)
non-variceal UGIB	mortality	<0.001	1.48	(1.26–1.75)
	rebleeding	0.11	1.12	(0.97–1.30)
	operation	0.003	1.24	(1.08–1.43)
	ICU treatment	<0.001	1.38	(1.20–1.59)
variceal UGIB	mortality	0.02	1.25	(1.03–1.52)
	rebleeding	0.03	1.20	(1.02–1.42)
	operation	0.04	1.20	(1.00–1.44)
	ICU treatment	0.02	1.34	(1.05–1.71)
bleeding peptic ulcers	mortality	<0.001	1.49	(1.21–1.84)
	rebleeding	0.42	1.08	(0.89–1.32)
	operation	0.01	1.25	(1.05–1.49)
	ICU treatment	<0.001	1.44	(1.19–1.74)

## Data Availability

The datasets used and/or analyzed during the current study are available from the corresponding author on reasonable request.
